# The Role of Magnetic Resonance Imaging in the Evaluation of Knee Pain

**DOI:** 10.7759/cureus.65898

**Published:** 2024-07-31

**Authors:** Neha D Shetty, Rajasbala P Dhande, Pratapsingh Parihar, Nikita Bora, Sheetal S Shelar

**Affiliations:** 1 Radiodiagnosis, Datta Meghe Institute of Higher Education and Research, Wardha, IND

**Keywords:** meniscus injury, anterior cruciate ligament (acl), chondromalacia pattela, non-traumatic knee pain, knee trauma

## Abstract

Objectives

This study aimed to characterize and compare the features of traumatic and non-traumatic lesions causing knee pain through magnetic resonance imaging (MRI).

Method

The study was conducted at a tertiary care center, with data sourced from patients visiting the outpatient and in-patient departments. It involved a descriptive cross-sectional research design focusing on patients referred for knee MRI scanning. The sample size was calculated using Cochran’s formula as 112 for symptomatic patients with knee pain with a 95% confidence interval. The MRI findings in 112 patients were analyzed and associated with a history of trauma.

Results

The average age recorded was 35.38 years. Females made up 41.07% (n=46) of the sample, while males accounted for 58.93% (n=66). Among the participants, the majority (n=82; 71.43%) had a history of trauma, and the most common MRI finding was joint effusion (n=74; 66.1%). The second most common was anterior cruciate ligament (ACL) injuries (n=71; 63.4%), followed by meniscus injury (n=40; 35.71%). The study confirms that those with history of trauma are at a higher risk (p<0.05) of sustaining injuries like meniscus and ACL tears, collateral ligament damage, bone contusions, chondromalacia patella, and joint effusion.

Conclusion

In conclusion, the consistency of our findings with existing studies reinforces the pivotal role of MRI in the evaluation of knee pain. Despite its limitations, including cost and accessibility, MRI remains a gold standard for diagnosing a wide range of knee pathologies, offering unparalleled detail and accuracy that significantly enhance clinical decision-making and patient outcomes.

## Introduction

Musculoskeletal (MSK) issues are a significant contributor to global morbidity, ranking fifth in disability-adjusted life-years (DALY), according to the 2017 Global Burden of Disease study [1]. They top the list of years lost to disability worldwide. Both men and women are equally impacted by MSK problems, which are believed to be a common underlying factor in physical limitations. The knee joint, a crucial synovial joint in the body, is one of the most extensive and intricate parts [2,3]. Serving as a hinge and weight-bearing joint, it is vulnerable due to the high demands placed on it. Comprising various bones, cartilage, muscles, tendons, and ligaments, the knee joint performs a wide array of functions. Excessive forces beyond its normal range can lead to bony or soft tissue injuries, with approximately 25% of the adults experiencing knee pain [4].

Knee pain, a prevalent issue accounting for a significant portion of musculoskeletal problems in primary care, has been on the rise in recent decades, regardless of age or BMI [5-7]. It is linked to reduced productivity and absenteeism, with a higher incidence among the elderly and females [8]. The causes of knee pain vary from meniscus tears to ligament pathologies, osteoarthritis, bone contusions, fractures, and inflammatory conditions [9]. Magnetic resonance imaging (MRI) plays a crucial role in diagnosing knee pain etiology, particularly in cases of suspected meniscus and cruciate ligament injuries. MRI is accurate, safe, non-invasive, and aids in avoiding unnecessary arthroscopies. It is recommended for individuals with persistent pain, swollen joints, or suspected tumors [10-13].

MRI has revolutionized musculoskeletal imaging since its introduction in the 1970s, with the knee being a commonly scanned joint. It is instrumental in evaluating meniscus and ligament injuries. Advanced MRI systems like 3 Tesla offer superior imaging quality and diagnostic accuracy, reducing scanning time. Studies have shown MRI to be highly reliable in evaluating knee conditions and guiding treatment decisions [14,15]. While traumatic knee pain is well-documented, chronic, non-traumatic, non-arthritic knee pain has received less attention in the medical literature. This research seeks to identify the underlying causes of chronic knee pain not related to trauma or arthritis [16-19].

## Materials and methods

The study was conducted at Acharya Bhave Rural Hospital, Sawangi, and Jawaharlal Nehru Medical College, and data were sourced from patients visiting the outpatient department (OPD) and in-patient department (IPD). It involved a descriptive cross-sectional research design focusing on patients referred for knee MRI scanning. For the study's sample selection, patients with knee joint pain were directed to Acharya Vinoba Bhave Rural Hospital in Sawangi. The sample size was calculated using Cochran's formula [20] as 112 for symptomatic patients with knee pain with a 95% confidence interval, an estimated proportion at 22%, and an estimation error of 8%. All individuals who underwent MRI specifically for the evaluation of knee pain were included in the study. Patients who have undergone knee surgery, pregnant individuals, those with metallic implants, pacemakers, or cochlear implants, as well as patients with claustrophobia or any other psychiatric abnormality, and those who are uncooperative were all excluded from participation in the study involving MRI scans for knee pain assessment.

All eligible patients were evaluated from July 2022 to July 2024. Once a patient met the research's eligibility requirements, they were given the study proforma to fill out before undergoing MRI scans using 3 Tesla machine. The procedure was explained to the patients. The patient was informed about the noise caused by gradient coils (heard once the patient was inside the magnet's bore) and the necessity to limit body movements during the scan duration.

In this study, all MRI scans of the knee were done utilizing a Philips 3 Tesla machine (Philips, Amsterdam, Netherlands). The MRI protocol includes the following sequences: coronal proton density (PD) with a slice thickness of 4mm and a skip of 0.5mm, sagittal T2-weighted image (T2WI) fast spin echo (FSE) with a slice thickness of 3mm, sagittal PD fast spin echo sequence (FSE) with a slice thickness of 4mm and a skip of 0.5mm, fat-suppressed FSE T2WI (axial, coronal, sagittal) with a slice thickness of 4mm and a skip of 0.5mm, short tau inversion recovery (STIR) FSE coronal with a slice thickness of 4mm and a skip of 0.5mm, STIR coronal with a slice thickness of 4mm and axial PD with a slice thickness of 6mm and a skip of 1.0mm. Contrast-enhanced study with gadolinium-based contrast agents was administered in the evaluation of cases of inflammatory conditions such as synovitis, neoplastic lesions, and infection.

A descriptive analysis was carried out by mean and standard deviation for quantitative variables and frequency and proportion for categorical variables. Non-categorical distributed quantitative variables were summarized using the median and interquartile range (IQR). Data was also represented using appropriate diagrams like bar diagrams, pie diagrams, and box plots. Chi-square test and Fisher's exact test [21] were used to compare categorical outcomes between study groups. A p-value of less than 0.05 was considered statistically significant. Data was analyzed using IBM SPSS Statistics, version 22 (IBM Corp., Armonk, NY, USA).

MRI images were analyzed as follows. The medial and lateral collateral ligament sprains were categorized based on their MRI characteristics: T2 hyperintense signal superficial and deep to the ligament without fiber discontinuity (Grade 1), partial fiber tearing (Grade 2), or complete ligament rupture (Grade 3). For analysis, all grades of ligament sprains were combined. Anterior and posterior cruciate ligament (ACL/PCL) sprains included both partial and complete tears. Patellar cartilage analysis was performed using the modified Outerbridge grading system, considering any cartilage damage (ranging from fraying/blistering to full-thickness loss) as cartilage abnormalities. Bone contusion was identified by marrow edema on fat-suppressed proton-density images, characterized by the absence of circumscribed borders or fracture lines.

## Results

A total of 112 subjects were included in the final analysis. The mean age in the study population was 35.38 ± 14.61 years, ranging between 13 and 70 years. Among the study population, 46 (41%) participants were female, and 66 (59%) were male.

The majority of the study population, which included 80 (71.43%) participants, had a history of trauma. In comparison, the rest (n=32, 28.6%) did not have a history of trauma. The MRI findings found in our study included ACL injuries, PCL injuries, meniscus injuries, joint effusion, collateral ligament injury, bone contusion, bone fractures, muscular edema, infection, osteoarthritis, neoplastic lesion, cystic lesions, and chondromalacia patella.

**Figure 1:**
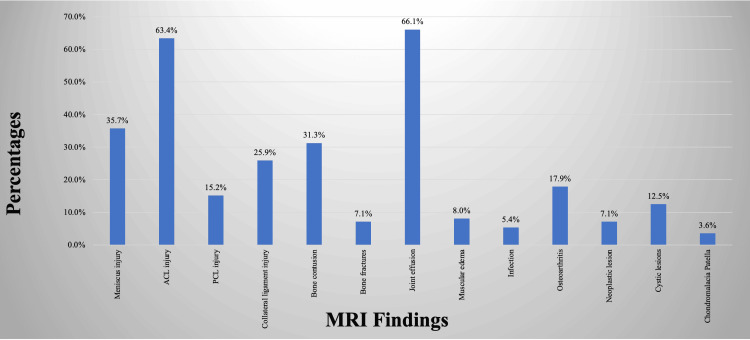
MRI findings in the study population and their frequency

The most commonly occurring finding, as depicted in Figure 1, was joint effusion, which was present in 74 (66.07%) scans, followed by ACL injuries in 71 (63.39%) and meniscus injuries in 40 (35.71%) out of a total of 112 scans. The correlation between the history of trauma and MRI findings was analyzed in our study in Table 1. The difference in MRI findings between the groups with and without a history of trauma with the presence of ACL injury, collateral ligament injury, and bone contusion was statistically significant (p<0.001).

**Table 1:** Correlation of MRI findings with history of trauma in the study population ACL: anterior cruciate ligament; PCL: posterior cruciate ligament.

MRI Findings	History of Trauma		P value
	Yes (N=80)	No (N=32)	
Meniscus injury	37 (46.25%)	3 (9.38%)	<0.001
ACL injury	63 (78.75%)	8 (25%)	<0.001
PCL injury	14 (17.5%)	3 (9.38%)	0.387
Collateral ligament injury	25 (31.25%)	4 (12.5%)	0.041
Bone contusion	32 (40%)	3 (9.38%)	0.002
Bone fractures	7 (8.75%)	1 (3.13%)	0.296
Chondromalacia patella	4 (5%)	1 (3.13%)	1.000
Joint effusion	61 (76.25%)	13 (40.63%)	<0.001
Muscular edema	9 (10.98%)	0 (0%)	0.048
Infection	0 (0%)	6 (20%)	0.003
Osteoarthritis	10 (12.5%)	10 (31.25%)	0.019
Neoplastic lesion	0 (0%)	8 (26.67%)	<0.001
Cystic lesions	7 (8.75%)	7 (21.88%)	0.058

Anterior cruciate ligament injuries

**Figure 2:**
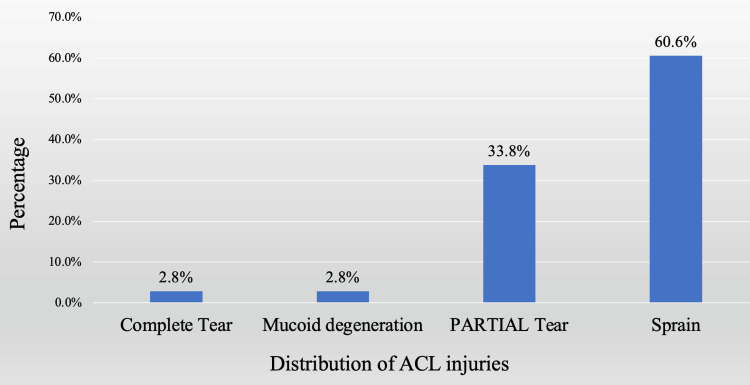
Distribution of the types of anterior cruciate ligament injury

Among the study population, 71 (63.39%) participants had ACL injuries, as depicted in Figure 2. Among the participants with ACL injury, two (2.82%) had a complete tear, two (2.82%) showed mucoid degeneration, 24 (33.80%) had a partial tear, and 43 (60.56%) had sprain.

Meniscus injuries

**Figure 3:**
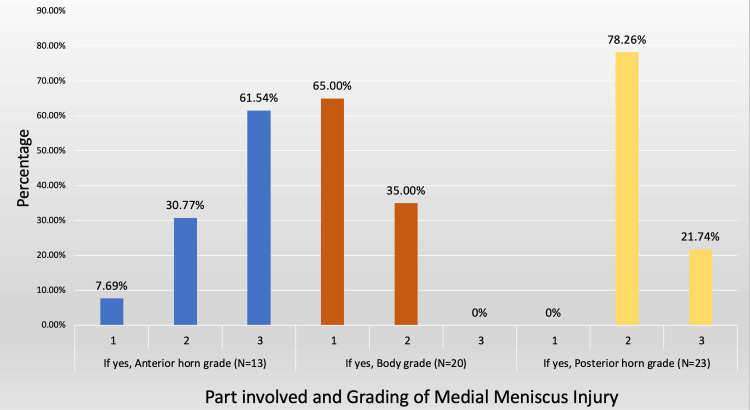
Grading of meniscus injury in each part of the medial meniscus along with their frequency

Among the study population, 40 (35.71%) participants had meniscus injury as depicted in Figure 3. Of the 40 participants, 25 (62.50%) had medial meniscus injury and 15 (37.50%) had lateral meniscus injury.

 

Medial Meniscus Injury

Among the participants with a medial meniscus injury, 13 (52%) had an injury in the anterior horn and 23 (92%) had a posterior horn and 20 (80%) participants had an injury in the body of medial meniscus. Out of 13 people with injury in the anterior horn of the medial meniscus, Figure 3 shows eight (61.54%) had Grade 3 injury, which was most common, followed by four (30.77%) with Grade 2 injury and one (7.69%) with Grade 1 injury. Out of 20 people with injury in the body of the medial meniscus, 13 (65%) had Grade 1 injury, which was the most common, followed by seven (35%) with Grade 2 injury. Of the 23 people with injury in the posterior horn of the medial meniscus, 18 (78.26%) had Grade 2 injury, followed by five (21.74%) with Grade 3 injury.

Lateral Meniscus Injury

Among the participants with a lateral meniscus injury, Table 2 shows the distribution of injury in different parts of the lateral meniscus and their respective grading, along with the frequency. A total of 11 (73.33%) participants had an injury in the anterior horn, among which eight (53.33%) had an injury in the posterior horn and five (33.33%) had an injury in the body of the lateral meniscus. Out of 11 with injury in the anterior horn of lateral meniscus, nine (81.82%) had Grade 2 injury, followed by one (9.09%) having Grade 3 injury and Grade 1 injury each. Out of five participants with injury in the body of the lateral meniscus, four (80%) had Grade 2 injury and one (20%) had Grade 1 injury. Out of eight with injury in the posterior horn of grade lateral meniscus, seven (87.50%) had Grade 2 injury and one (12.50%) had Grade 1 injury.

**Table 2:** Grading of meniscus injury in each part of the lateral meniscus along with their frequency

Lateral Meniscus Injury	Frequency	Percentage (%)
Anterior horn (N=15)		
Yes	11	73.33
No	4	26.67
Yes in anterior horn (N=11)		
Grade 1	1	9.09
Grade 2	9	81.82
Grade 3	1	9.09
Body (N=15)		
Yes	5	33.33
No	10	66.67
Yes in body (N=5)		
Grade 1	1	20.00
Grade 2	4	80.00
Grade 3	0	0
Posterior horn (N=15)		
Yes	8	53.33
No	7	46.67
Yes in the posterior horn (N=8)		
Grade 1	1	12.50
Grade 2	7	87.50
Grade 3	0	0

Cystic lesions

Out of the MRI scans examined, only 14 (12.50%) were found to have cystic lesions. The type of cystic lesions in these scans was determined as follows: Baker's cyst was seen in 11 (78.57%) participants. Ganglion cyst was noted in one (7.14%) and parameniscal cyst in two (14.29%) participants.

Chondromalacia patella

Out of the 112 participants, five (4.46%) had chondromalacia patella, as seen in Table 3. Among the four individuals diagnosed with chondromalacia patella, the distribution of the condition by severity showed one (20%) had Grade 1, indicating mild cartilage damage, two (40%) had Grade 2, indicating moderate cartilage damage, and one each (20%) had Grade 3 and Grade 4, indicating more severe damage to the cartilage.

**Table 3:** Frequency and grading of chondromalacia patella

Chondromalacia Patella	Frequency	Percentages
Yes	5	4.46%
No	107	95.54%
If yes, Grade of chondromalacia patella (N=5)	Frequency	Percentages
Grade 1	1	20%
Grade 2	2	40%
Grade 3	1	20%
Grade 4	1	20%

## Discussion

MRI is an essential technique for evaluation in cases of acute and chronic knee pain because it can identify a wide variety of diseases, from meniscal injuries and ligament rips to bone marrow abnormalities and cartilage deterioration. MRI provides accurate and timely diagnosis, which is helpful in creating treatment plans and is essential for tracking the effectiveness of therapeutic therapies. In the present study, joint effusion was the most common finding in 74 (66%) subjects. Similar to the present study, Rana et al. [19] observed that joint effusion was the most common MRI finding in patients with painful knee joints, followed by meniscus injury and ACL injury. The mean age was 35.38 years in the present study, and the majority of participants were male (n=66, 59%). Van Oudenaarde et al. [22] studied 174 adults, aged between 18 and 45 years, with a history of recent trauma (less than six months) to the knee and presenting for MRI and found that the majority (71%) of study subjects were male similar to the present study.

Our study revealed that 71 (63.39%) participants had an ACL injury. Out of them, only two had a complete tear. On the other hand, out of the 17 (15.18%) participants with PCL injury, four had complete tears in the present study. Mattoo et al. [23] observed that 67% of the studied patients had partial ACL injury in their study. The findings of the present study are comparable with their study. But Rana et al. [19] observed joint effusion as the most common pathology in patients, followed by meniscus injury and anterior cruciate ligament tear. Bansal et al. [16] observed that tear was the most prevalent ACL pathology, with a majority of the cases being acute. These variations could be due to the type of work, the type of trauma involved, and the age/gender distribution across studies.

In the present study, 40 (35.71%) participants had meniscus injury; of these, 25 (62.5%) subjects had medial and 15 (37.5%) had lateral meniscus injury. Among the subjects with medial meniscus injury, the majority had posterior horn injury, followed by injury in the body of the medial meniscus in the present study. With regards to lateral meniscus injury, the majority, eight (53.33%) had an injury in the anterior horn, followed by five (33.33%) having an injury in the posterior horn. The meta-analysis of meniscal tears done by Wang et al. [24] included a detailed assessment of MRI's diagnostic accuracy. They confirmed that MRI is a reliable diagnostic tool for meniscal tears, reporting good specificity and sensitivity for both medial and lateral tears. These MRI findings show that excellent diagnostic accuracy in detecting meniscal tears is crucial for creating successful treatment regimens. The present study findings were consistent with other studies. Mattoo et al. [23] observed medial meniscal lesions in 49% of the studied patients and lateral meniscal lesions in 16% of them. In the present study, 14 (12.5%) subjects had cystic lesions, with Baker's cyst being the most commonly observed cyst among them. Bansal et al. [16] observed that the most prevalent cystic lesion was the popliteal cyst, linked with effusions and meniscal tears.

The present study found a strong correlation between the individual's history of trauma and several MRI findings. People with a history of trauma are more likely to have injuries such as meniscus and ACL tears, collateral ligament damage, bone contusions, joint effusion, chondromalacia patella, and muscular edema. Conversely, people without a history of trauma were more likely to have infections, osteoarthritis, and neoplastic lesions. These results highlight the importance of taking the history of trauma into account when diagnosing, evaluating, and treating knee injuries.

## Conclusions

To sum up, MRI is a vital modality for assessing knee pain because it provides unparalleled precision and detail in diagnosing a broad range of knee disorders. It is an essential tool in modern orthopedic practice because it improves diagnostic accuracy, guides treatment plans, and tracks recovery. However, the high cost and restricted availability of MRI facilities can act as obstacles. Furthermore, not every episode of knee pain requires an MRI, especially if the cause is obvious and can be identified by clinical examination. Notwithstanding these drawbacks, MRI offers significant advantages in the management of knee pain, especially in complex instances where accurate imaging is essential for the best possible patient outcomes. In conclusion, the consistency of our findings with existing studies reinforces the pivotal role of MRI in evaluating knee pain. Despite its limitations, including cost and accessibility, MRI remains a gold standard for diagnosing a wide range of knee pathologies. It offers unparalleled detail, significantly enhancing clinical decision-making and patient outcomes.
